# Diagnostic and Monitoring Potential of Sputum-Derived miRNAs in Patients with Chronic Obstructive Pulmonary Disease

**DOI:** 10.3390/ijms27146218

**Published:** 2026-07-12

**Authors:** Federica Tonon, Domenico Tierno, Alice Biasin, Marco Confalonieri, Barbara Ruaro, Erminio Murano, Davide Manca, Chiara Grassi, Michela Abrami, Serena Bonin, Bruna Scaggiante, Mario Grassi, Gabriele Grassi

**Affiliations:** 1 Department of Medical, Surgical and Health Sciences, Cattinara University Hospital, Trieste University, Strada di Fiume 447, I-34149 Trieste, Italy; federica.tonon@asugi.sanita.fvg.it (F.T.); domenico.tierno@units.it (D.T.); alice.biasin@phd.units.it (A.B.); mconfalonieri@units.it (M.C.); barbara.ruaro@units.it (B.R.); sbonin@units.it (S.B.); 2 PROTOS Research Institute, Via del Botro 10/8, I-34149 Trieste, Italy; erminio.murano@protos-institute.org; 3 Department of Chemistry, Materials, and Chemical Engineering “Giulio Natta” Milan Politecnico, I-20151 Milan, Italy; davide.manca@polimi.it; 4 Degree Course in Medicine, University of Trieste, I-34149 Trieste, Italy; chiara.grassi2@studenti.units.it; 5 Department of Engineering and Architecture, Trieste University, Via Valerio 6, I-34127 Trieste, Italy; michela.abrami@dia.units.it; 6 Department of Life Sciences, University of Trieste, I-34127 Trieste, Italy; bscaggiante@units.it

**Keywords:** miRNA, COPD, sputum

## Abstract

Chronic obstructive pulmonary disease (COPD) is a heterogeneous and progressive respiratory disorder characterized by airflow limitation, chronic inflammation, and structural lung alterations. Despite advances in clinical monitoring, current approaches such as spirometry, symptom scores, and imaging remain limited in capturing disease complexity and early pathophysiological changes. In this context, microRNAs (miRNAs) have emerged as promising molecular biomarkers due to their involvement in key inflammatory and immune pathway regulation. Sputum is gaining attention as a non-invasive and informative matrix for studying COPD. As a surrogate of airway mucus, sputum reflects local pathological processes, including inflammation and tissue damage, and contains biomarkers such as miRNAs. Compared to circulating miRNAs, sputum-derived miRNAs appear to more accurately represent lung-specific alterations. In this review, we focus on works published so far about the identification of miRNAs in COPD sputum. Several miRNAs have been associated with COPD diagnosis, severity, and exacerbations. These findings, including the possible correlation of some miRNAs with sputum biophysical properties, support the value of integrated biomarker approaches. While further large-scale and standardized studies are required to validate the role of COPD miRNAs assessed in sputum, their evaluation in sputum represents a promising tool for improving the diagnosis, phenotyping and monitoring of COPD.

## 1. Introduction

### 1.1. COPD

Chronic obstructive pulmonary disease (COPD) is a progressive respiratory disorder characterized by persistent airflow limitation and chronic inflammation of the airways and lung parenchyma. It represents a major global health burden and is currently one of the leading causes of morbidity and mortality worldwide [1,2,3,4]. COPD encompasses a spectrum of pathological conditions, primarily chronic bronchitis and emphysema, which frequently coexist and contribute to disease severity.

The principal risk factor for COPD is long-term exposure to harmful particles and gases, with cigarette smoking being the most significant contributor. However, environmental and occupational exposures, such as air pollution, biomass fuel combustion, and industrial irritants, also play a crucial role [1,5]. In addition, genetic predisposition, impaired lung development, and aging processes may influence individual susceptibility to the disease [6,7].

From a pathophysiological perspective, COPD is characterized by a chronic inflammatory response involving both innate and adaptive immune mechanisms [8]. This leads to structural and functional alterations, including goblet cell hyperplasia, mucus hypersecretion, airway wall thickening, and fibrosis. Concurrently, destruction of alveolar structures results in loss of elastic recoil and impaired gas exchange, hallmarks of emphysema. These changes ultimately result in airflow limitation, air trapping, and progressive decline in lung function.

Clinically, COPD presents with chronic cough, sputum production, and dyspnea, which progressively worsen over time. Acute exacerbations, defined as episodes of symptom worsening beyond normal variation, are often triggered by infections or environmental factors and are associated with accelerated lung function decline, reduced quality of life, and increased mortality [9].

The management of COPD primarily relies on pharmacological therapies aimed at alleviating symptoms, reducing the frequency of exacerbations, and enhancing patients’ quality of life. The cornerstone treatments include bronchodilators—such as long-acting muscarinic antagonists (LAMAs) like Tiotropium, and long-acting β2-agonists (LABAs) such as Salmeterol and Formoterol—and their combination therapies (e.g., dual LAMA/LABA inhalers). In addition, inhaled corticosteroids (ICSs) are often prescribed, particularly for patients experiencing frequent exacerbations and exhibiting eosinophilic inflammation [1]. Recently, biological therapies have emerged as promising options for certain COPD phenotypes, especially in patients with eosinophilic inflammation. Monoclonal antibodies targeting specific inflammatory mediators—such as mepolizumab and benralizumab, which target interleukin-5 (IL-5)—have shown efficacy in reducing exacerbation frequency in eosinophilic COPD [10,11]. These biologics can modulate airway inflammation more precisely than traditional therapies, providing tailored treatment options for selected patients. While their use is more established in asthma, growing evidence supports their role in managing a subset of COPD patients with elevated eosinophil counts, offering potential for disease modification beyond symptomatic relief [12]. Current management strategies focus on symptom control and prevention of exacerbations through pharmacological therapies such as bronchodilators and inhaled corticosteroids, as well as non-pharmacological interventions including smoking cessation and pulmonary rehabilitation [1].

While these therapies have demonstrated significant benefits in symptom control and exacerbation prevention, they also present notable limitations. Primarily, current pharmacological options are limited to symptomatic relief and do not modify the underlying disease progression [1]. Moreover, there is considerable heterogeneity in patient response, which complicates prediction of therapeutic efficacy and highlights the need for better phenotypic stratification [1]. Additionally, existing biomarkers lack the sensitivity and specificity required to reliably predict exacerbation risk or to monitor early molecular changes associated with disease worsening [10]. Imaging and functional assessments, although valuable, often fall short in detecting early molecular-level changes or in evaluating treatment responses at the level of airway inflammation [10].

Given these constraints, there is a pressing need for novel biomarkers—such as sputum-derived microRNAs—that can provide deeper insights into the molecular mechanisms underlying COPD. These biomarkers could enable early detection of disease progression, facilitate personalized therapeutic approaches—including the potential use of biologics—and complement existing clinical and functional assessments. Ultimately, integrating such molecular tools could lead to more precise and dynamic management strategies for COPD patients. This need is underscored by the fact that current approaches do not fully halt disease progression, highlighting the importance of improving our understanding of the underlying molecular mechanisms.

### 1.2. Current Monitoring Approaches

Effective monitoring of COPD is crucial for evaluating disease progression, guiding therapeutic decisions and reducing the risk of exacerbation. Current strategies rely on a combination of clinical, functional, imaging, and biomarker-based approaches, each contributing complementary information [10].

A central component of COPD monitoring is spirometry, particularly the measurement of forced expiratory volume in one second (FEV_1_), which is used for diagnosis and disease staging according to GOLD (Global Initiative for Chronic Obstructive Lung Disease) guidelines [11]. However, FEV_1_ correlates only modestly with symptoms, exacerbation risk, and quality of life, limiting its ability to fully capture disease complexity [12,13]. Clinical assessment includes evaluation of symptoms, exacerbation history, and health status, often using validated tools such as the COPD Assessment Test (CAT) [14] and the modified Medical Research Council (mMRC) dyspnea scale [15]. Although these measures are essential, they are inherently subjective and influenced by patient perception and reporting variability.

Imaging techniques, particularly high-resolution computed tomography (HRCT), provide detailed structural information, including emphysema distribution and airway remodeling [16]. However, their routine use is limited by cost, radiation exposure, and lack of standardized quantitative thresholds.

The use of biomarkers is an expanding field in COPD monitoring. Circulating markers such as C-reactive protein and fibrinogen reflect systemic inflammation and are associated with disease severity and outcomes [17]. More recently, analysis of sputum has gained interest, as it reflects local airway inflammation. Serum and sputum biomarkers, such as microRNAs (miRNAs), may provide insight into lung-specific pathological processes, improving disease phenotyping [18].

Despite these advances, significant limitations remain. COPD is a highly heterogeneous disease, and no single parameter adequately reflects its pathophysiology. Many current tools are insufficiently sensitive to detect early changes or predict exacerbations accurately. Furthermore, methodological variability, comorbidities, and environmental factors may influence measurements and reduce reliability. Consequently, there is a growing need for integrated and multimodal monitoring approaches combining clinical, functional, imaging, and molecular data.

### 1.3. miRNAs

To date, a wide variety of non-coding RNAs (ncRNAs) have been identified, including microRNAs (miRNAs) and long non-coding RNAs (lncRNAs) [19]. ncRNAs play a crucial role in the regulation of gene expression by interacting with their target RNAs. Moreover, they can also interact with one another, forming a highly complex regulatory network that finely controls gene expression in human cells. Among these, miRNAs have been the most extensively studied to date.

**Figure 1:**
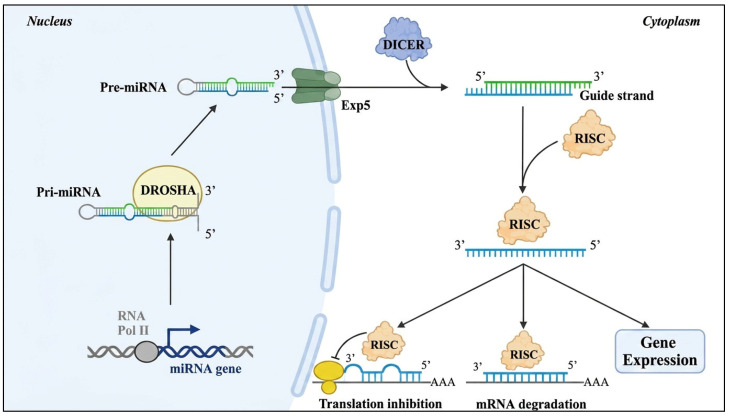
miRNA biogenesis and functions. In the nucleus, primary microRNA (pri-miRNA) transcripts are processed by the Drosha enzyme into precursor miRNAs (pre-miRNAs). These precursors are then transported to the cytoplasm by Exportin-5 (Exp-5). Once in the cytoplasm, Dicer cleaves the pre-miRNAs to generate double-stranded mature miRNAs. The guide (antisense) strand is incorporated into the RNA-induced silencing complex (RISC), which facilitates binding to target mRNAs. Depending on the degree of sequence complementarity, this interaction results in either translational repression (imperfect pairing) or mRNA degradation (perfect or near-perfect pairing). This figure was created in BioRender. Scaggiante, B. (2026) https://BioRender.com/1omfo82.

miRNAs are a class of short, double-stranded non-coding RNAs that were first identified as key regulators of human gene expression [20]. miRNAs are primarily transcribed in the nucleus as long primary transcripts, named pri-miRNAs, originating from either coding or non-coding genomic regions (Figure 1). These transcripts undergo sequential processing, initially mediated by the RNase III enzyme Drosha, which cleaves pri-miRNAs into precursor miRNAs (pre-miRNAs) [21].

Pre-miRNAs are subsequently exported to the cytoplasm via Exportin 5, where they are further processed by the DICER enzyme into mature miRNA duplexes of approximately 22 nucleotides, characterized by 3′ overhangs [22]. One strand of the mature miRNA duplex, the guide strand, is incorporated into the RNA-induced silencing complex (RISC), enabling sequence-specific interaction with target mRNAs through base pairing. Canonically, effective translational repression requires complementarity involving nucleotides 2–8 of the miRNA seed region at the 5′ end. Depending on the degree of complementarity, miRNA binding can result in translational inhibition or target mRNA degradation, most commonly occurring at the 3′ untranslated region. Moreover, miRNA function is not restricted to gene silencing, as emerging evidence indicates that miRNAs can, in selected contexts, enhance gene expression through direct or indirect regulatory mechanisms [23].

The biological relevance of miRNAs is further amplified by the complexity of their regulatory networks. Individual miRNAs can target multiple mRNAs, while single mRNA transcripts can be modulated by multiple miRNAs [24]. Adding another level of complexity, miRNAs can circulate in the bloodstream encapsulated within extracellular vesicles, thereby functioning as intercellular signaling molecules with hormone-like properties. In the context of COPD, miRNAs have been extensively implicated in disease pathogenesis and progression and are increasingly recognized as promising diagnostic and prognostic biomarkers [18,25,26].

### 1.4. Extracellular Vesicles

**Figure 2:**
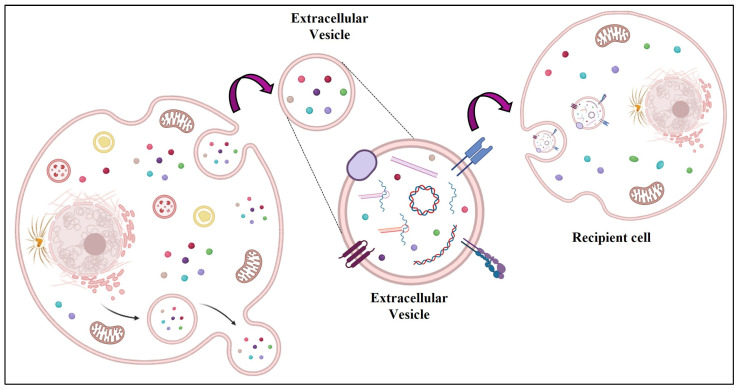
Extracellular vesicles (EVs) are a heterogeneous population of lipid bilayer-enclosed particles that are produced and secreted by various cell types into the extracellular environment. These vesicles carry a diverse array of bioactive molecules, including proteins, lipids, and nucleic acids. It is now well established that EVs can also contain microRNAs and mediate their transfer to distant recipient cells under both physiological and pathological conditions. This figure was created in BioRender. Scaggiante, B. (2026) https://BioRender.com/usd4ww8.

Extracellular vesicles (EVs) have emerged as key mediators of intercellular communication, particularly following the recognition that ncRNAs, including miRNAs, can circulate systemically encapsulated within these vesicles and exert hormone-like functions [27,28]. EVs comprise a heterogeneous population of lipid bilayer-enclosed particles released by virtually all cell types into the extracellular space and are detectable in multiple biological fluids, including blood [29]. EVs are commonly classified according to their size and biogenetic origin into small EVs, usually <200 nm; medium/large EVs, usually >200 nm; microvesicles; and apoptotic bodies [30]. Although the molecular mechanisms governing EV biogenesis are not yet fully elucidated, current evidence supports two principal pathways: the formation of intraluminal vesicles within the endocytic system and the direct outward budding of vesicles from the plasma membrane (Figure 2).

**Figure 3:**
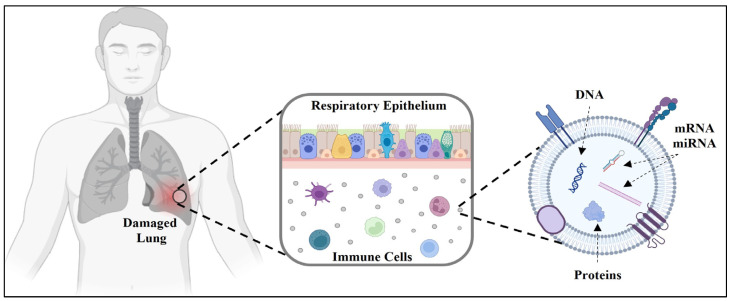
Lung-derived extracellular vesicles (EVs) can be released into the local microenvironment or enter the circulation, enabling intercellular communication both within the lung and with distant organs. Through the transfer of their molecular cargo, EVs contribute to the regulation of physiological processes such as tissue homeostasis and immune responses, as well as pathological conditions, including inflammation, infection, and lung diseases. This figure was created in BioRender. Scaggiante, B. (2026) https://BioRender.com/w4wzn1w.

The role of EVs has also been recognized in COPD [30]. Epithelial cells, endothelial cells, and diverse immune cell populations within the lung contribute to the production of EVs in the pulmonary microenvironment (Figure 3).

These EVs carry molecular cargo that reflects their cellular origin. Under physiological conditions, EVs play a critical role in maintaining pulmonary homeostasis by modulating immune responses and promoting tissue repair processes. In COPD, both airway epithelial cells and resident immune cells exhibit enhanced EV release. Quantitative analyses have demonstrated that the proportion of EVs within the 150–200 nm size range is approximately twofold higher in the sputum of COPD patients compared to healthy controls [31]. Furthermore, sputum samples from COPD patients contain elevated levels of EV-associated proteins, indicating a disease-specific alteration in EV composition and abundance relative to non-diseased conditions. In recent years, increasing attention has been directed toward the role of EV-associated miRNAs in COPD. Accumulating evidence indicates that miRNAs selectively packaged into EVs contribute to the pathophysiology of COPD and represent valuable elements for both diagnostic and therapeutic applications [18].

### 1.5. Sputum

#### 1.5.1. Sputum vs. Lung Mucus

While miRNAs are mostly investigated in blood, other biological fluids are attracting increasing interest. In the field of COPD, sputum is emerging as an attractive biological sample. Sputum is widely regarded as a valid surrogate of lung mucus because it originates from the lower respiratory tract and retains the main structural, biochemical, and functional characteristics of mucus [32].

**Figure 4:**
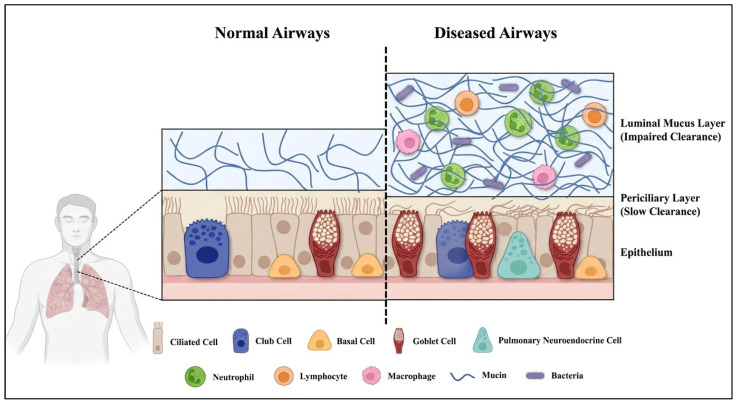
Schematic representation of the airway epithelium under normal and diseased conditions. In normal airways, a well-organized epithelial layer composed of ciliated, club, basal, goblet, and pulmonary neuroendocrine cells is covered by a balanced mucus layer and an underlying periciliary layer that supports efficient mucociliary clearance. In diseased airways, structural and functional alterations occur, including increased mucus production and accumulation in the luminal layer, reduction of the periciliary layer, impaired clearance, and changes in epithelial cell composition. The diseased microenvironment is also characterized by the presence of inflammatory cells, such as neutrophils, macrophages, and lymphocytes, along with microbial components, contributing to inflammation and disrupted airway functions. This figure was created in BioRender. Scaggiante, B. (2026) https://BioRender.com/kyil1ue.

Physiologically, mucus is continuously produced by goblet cells and submucosal glands lining the bronchi and lungs, forming a protective viscoelastic layer that traps inhaled particles and microorganisms and is cleared via mucociliary transport (Figure 4). Epithelial cells are interconnected by tight junctions, which maintain an impermeable barrier essential for airway protection. The mucus layer is physically separated from the epithelial surface by an aqueous periciliary layer that permits effective ciliary beating and mucociliary clearance. Mucus is composed of water, mucin glycoproteins, proteins, lipids, electrolytes, and immune molecules [33].

Among other alterations, one typical feature of COPD mucus is the modification of ciliated cells [8]. The periciliary layer becomes diminished, leading to ciliary crowding and impaired motility. Additionally, disruption of epithelial tight junctions compromises barrier integrity. Collectively, these alterations contribute to defective mucociliary clearance and increase susceptibility to airway inflammation and injury.

Sputum contains similar mucus constituents along with cells, such as neutrophils and macrophages, DNA, microorganisms, and inflammatory mediators [34]. Thus, sputum is considered a “window into the lower respiratory tract”, as it provides direct information about infection, inflammation, and immune responses occurring in the lungs. Additionally, sputum contains EVs [30,31,35,36], which are known to contain various biological molecules, including miRNAs.

#### 1.5.2. Advantages and Disadvantages of Sputum as a Biological Source of miRNAs

There are several advantages in the use of sputum as a biological sample source in COPD monitoring (Table 1). First, sputum represents a non-invasive and repeatable biological sample, unlike bronchoscopy or bronchoalveolar lavage, which require more complex medical organization. Second, because of its composition, it is suitable for biochemical, microbiological, rheological, and cellular analyses. Third, it can reflect early local inflammatory changes and exacerbation-related signals. Fourth, compared to miRNAs measured in blood, miRNAs in sputum are more likely to directly depend on local pathophysiological events, such as cytokine expression, epithelial barrier function, and oxidative stress responses [34]. The measurement of miRNAs in blood can be influenced by confounding variables, such as comorbidities, including cardiovascular disease and diabetes, systemic medications, and systemic inflammation. Finally, treatments such as inhaled corticosteroids or bronchodilators act locally; therefore, changes in sputum miRNAs may better reflect treatment response in the lungs than blood miRNAs.

Despite the above advantages, miRNA quantification in sputum also has some drawbacks. Pre-analytical workflows are not standardized, and they can impact downstream analyses [37]. Obtaining sputum of adequate quality can be technically demanding and may not always be feasible in all patients. Sputum may be altered by coughing and mixing with saliva. Additionally, there is lower standardization in miRNA quantification compared to blood-based assays.

#### 1.5.3. Sputum Collection

Sputum samples may be collected either spontaneously or through induction [32]. Spontaneous expectoration depends on patient effort, whereas induced sputum is obtained via inhalation of nebulized isotonic or hypertonic saline solution, followed by voluntary coughing to obtain airway secretions [38]. As inhalation of saline may provoke bronchoconstriction, appropriate safety precautions must be implemented. Pretreatment with inhaled salbutamol is recommended, with a standard dose of 200 µg administered prior to saline inhalation. The induction procedure should be conducted using a sterile, freshly prepared saline solution. A hypertonic saline solution, usually 4.5% sodium chloride, is generally recommended for routine use, as it typically yields a greater sputum volume compared to isotonic saline. However, despite this difference in sample quantity, no significant differences in cellular composition have been observed between sputum samples obtained using isotonic versus hypertonic saline solutions. Both spontaneous and induced sputum collection are susceptible to contamination by saliva and epithelial cells. However, induced sputum samples generally demonstrate improved quality with respect to overall cell viability [39]. Finally, the use of saline solution can alter the viscoelastic properties of mucus, making it unsuitable for physical analysis based, for example, on rheology tests [40].

## 2. Detection of miRNA in COPD Sputum

The quantification of miRNAs in COPD sputum represents a relatively novel approach for disease monitoring. This likely accounts for the limited number of studies identified in the literature. Nevertheless, this area shows considerable promise for improving COPD stratification and monitoring. The findings described are summarized in Table 2. The methodology used to retrieve publications (PubMed) was based on the keywords “miRNA,” “sputum,” and “COPD.” No restrictions were applied regarding the publication date. This approach ensured a comprehensive retrieval of the literature related to the topic, giving an unbiased overview of the available literature.

Kiszałkiewicz et al. quantified miRNA-155 levels in induced sputum and peripheral blood lymphocytes from forty COPD patients compared with a control group of twenty subjects [41]. miRNA-155 is involved in the modulation of immune responses and inflammation, promoting T cell-dependent tissue inflammation [42]. Apparently, miRNA-155 was quantified in the whole sputum and not in isolated exosomes. There were no statistically significant differences among COPD patient groups classified according to GOLD A–D categories. However, a significant difference was observed between current smokers and ex-smokers, with upregulation in current smokers. Notably, there was no statistically significant difference between these groups when miRNA-155 was quantified in peripheral blood lymphocytes. Although the low number of patients is a limitation, this investigation suggests that sputum may represent an attractive source to evaluate inflammatory status in COPD, at least concerning the differentiation between current and ex-smokers. The data reported by Conickx et al. indicated that miRNA-155 and miRNA-135b are significantly upregulated in COPD patients compared with controls when quantified in bronchial biopsies [43]. miRNA-135b is involved in the pathoetiology of several neoplastic and non-neoplastic conditions [44]. This observation strengthens the findings of Kiszałkiewicz et al., supporting miRNA-155 as an attractive candidate to monitor lung inflammation in COPD [41]. The authors further reinforced the potential monitoring utility of miRNA-155 by showing that it was upregulated in cell-free bronchoalveolar lavage supernatant of mice exposed to cigarette smoke for 24 weeks. Moreover, miRNA-155 upregulation directly correlated with the number of B cells in the lung, supporting its pro-inflammatory role.

Additional evidence for miRNA-155 involvement in COPD comes from Kotsiou et al. [45]. In induced sputum from twenty clinically stable COPD patients and ten age-matched healthy controls, the authors observed downregulation of miRNA-155, miRNA-126, and miRNA-146a, and upregulation of miRNA-21 and miRNA-223. These miRNAs are all involved in COPD pathogenesis [18]. Notably, the finding of miRNA-155 downregulation seems to contrast with the work of Conickx et al., where this miRNA was found to be upregulated in COPD patients [43]. However, in the study by Kotsiou et al., miRNA-155 was measured in sputum-derived exosomes, whereas in the work of Conickx et al., it was quantified in bronchial biopsies. Additionally, the small number of patients considered in both studies may contribute to the discrepancy, as the two COPD populations may not have been homogeneous. Despite this, Kotsiou et al. [45] showed that miRNA-21 correlated with symptom burden and that miRNA-155 exhibited the best diagnostic performance for COPD detection, which was further improved when combined with miRNA-126 and miRNA-146a. For disease stratification, miRNA-126 was the most effective in discriminating mild from moderate-to-severe forms of COPD. Thus, the non-invasive quantification in sputum of the combination of miRNA-155, miRNA-126, and miRNA-146a may improve COPD diagnostic detection and staging accuracy.

The deregulation of miRNA-126, 146a, 21 and 223 reported by Kotsiou et al. [45] has also been detected in biological samples different from sputum. miR-126 was reduced in peripheral blood mononuclear cells (PBMCs) isolated from patients experiencing an acute exacerbation of COPD, in comparison to those obtained from patients with stable COPD and healthy controls [46]. Nadi et al. [47] observed a significant downregulation in the expression of miR-146a in PBMCs isolated from COPD patients. The down-regulation, which was more evident in the presence of cigarette smoke exposure, was present not only in the patient groups but also in healthy individuals exposed to smoking. The upregulation of miRNA-21 has been documented in the lung tissue of COPD smokers [48]. This upregulation has been shown to induce autophagy and emphysema. Finally, an increased expression of miRNA-223 was identified in the lung tissue of patients diagnosed with COPD, and this was found to be associated with elevated levels of neutrophilic-derived inflammation in response to smoking [49]. Taken together, these findings indicate a consistent correlation between these miRNAs quantitated in sputum and those measured in other biological specimens in the context of COPD.

miRNA-145 is involved in the regulation of airway smooth muscle function, which contributes to airway remodeling during chronic inflammation in COPD [50]. miRNA-338 is implicated in the control of cellular differentiation, apoptosis, and tissue degeneration [51]. Lacedonia et al. [52] quantified miRNAs in spontaneous sputum samples from thirty-one consecutive COPD patients. As the analysis was conducted on the sputum supernatant after brief centrifugation, the results likely reflect extracellular miRNAs, encompassing both exosomal and cell-free fractions. Notably, miRNA-145 expression was elevated in COPD patients compared to seven healthy non-smoking controls, with comparable expression levels observed in both sputum and serum. miRNA-338 was also upregulated in COPD sputum, but its levels were higher in sputum than in peripheral blood samples, while no differences were found in healthy subjects. In line with the above observation, increased levels of miR-145 were identified in airway smooth muscle (ASM) cells isolated from COPD patients when compared to those obtained from healthy nonsmokers and healthy smokers [50]. In contrast, miRNA-338 was found to be down-regulated in bronchoalveolar lavage fluid EVs from patients diagnosed with mild-to-moderate COPD in comparison to those from healthy ex-smokers [53]. Although it is difficult to explain the discrepancy with the findings of Lacedonia et al. [52], it is possible that the use of different biological samples, spontaneous sputum versus EVs isolated from bronchoalveolar lavage fluid, may have influenced the quantification.

Van Pottelberge et al. considered seventy-three subjects divided into never-smokers without airway obstruction, current smokers without airway obstruction, and currently smoking patients with COPD [54]. As the analysis was conducted on the sputum supernatant after brief centrifugation, the results likely reflect extracellular miRNAs, encompassing both exosomal and cell-free fractions. In induced sputum, the authors measured miRNA levels and found that let-7c and miRNA-125b levels were significantly lower in the sputum of currently smoking patients with COPD compared with never-smokers without airflow limitation. Moreover, let-7c and miRNA-125b levels were not different between ex-smoking patients with COPD and subjects without airflow limitation. This suggests that, at least in this cohort, smoking status rather than COPD itself determines let-7c and miRNA-125b downregulation. Subsequent analyses revealed that Let-7 expression was downregulated in peripheral lung tissues, primary bronchial epithelial cells, and serum collected from COPD patients when compared with that in non-smokers and smokers [55]. Most recently, the upregulation of miR-125b, analyzed in serum-derived EVs, was identified as a potential mediator of frailty-related comorbidities in patients with obstructive pulmonary diseases [56].

miRNA-181a plays a critical role in COPD-induced airway inflammation [57]. Tuersun et al. [58] measured miRNA-181a level in induced sputum from twenty COPD patients and twenty healthy controls. It remains unclear whether miRNA quantification was performed on the sputum supernatant (i.e., exosomal miRNAs) following centrifugation or on the cellular pellet (cell-associated miRNAs). The authors found that miRNA-181a expression was upregulated in COPD patients compared to controls. In parallel, the levels of BPIFB4 and the long non-coding RNA LncTUG1 were downregulated. BPIFB4, mainly concentrated in the upper respiratory tract and the proximal ends of the trachea, has anti-inflammatory properties; moreover, its expression in lung tissue of COPD patients is downregulated [59,60]. LncTUG1 belongs to the class of long non-coding RNAs. LncRNAs are single-stranded RNA molecules longer than 200 nucleotides. They closely resemble mRNAs, as they are transcribed by RNA polymerase II and typically undergo canonical post-transcriptional processing, including splicing, 5′ capping, and 3′ polyadenylation. In contrast to mRNAs, however, lncRNAs lack a translated open reading frame and do not encode proteins [19]. LncTUG1 is involved in regulating immune responses and inflammatory signaling pathways [61]. Tuersun et al. [58] showed that downregulation of LncTUG1 resulted in increased miRNA-181a, which in turn downregulated BPIFB4. Thus, the downregulation of LncTUG1 promotes inflammation by reducing BPIFB4 expression through miRNA-181a. The work of Tuersun et al. supports the evidence that sputum represents a valuable source of biomarkers, including but not limited to miRNAs. In contrast to the findings of Tuersun et al. [58], miR-181a expression was significantly downregulated in PBMCs from patients with stable COPD, as well as those experiencing acute exacerbations, compared with healthy controls [62]. These results support the notion that the regulation of this microRNA may vary across different biological compartments.

Zhu et al. observed that the levels of miRNA-122 and miRNA-30a were significantly decreased in induced sputum and plasma of COPD patients during exacerbation compared to non-exacerbated subjects [63]. It remains unclear whether miRNA quantification was performed on the sputum supernatant (i.e., exosomal miRNAs) following centrifugation or on the cellular pellet (cell-associated miRNAs). In parallel, IL-17A expression was elevated in plasma, peripheral blood monocytes, and sputum of exacerbated COPD patients compared to non-exacerbated patients. The authors also showed that IL-17A is a target of miRNA-122 and miRNA-30a, thus proving a functional connection between these miRNAs and IL-17A in exacerbated COPD patients. Notably, IL-17A favors the production of lymphocytes, as well as the proliferation of autoreactive B and T cells, thereby promoting inflammation and indirectly contributing to COPD pathophysiology [64]. Interestingly, the authors showed that, in smoke-induced COPD mice, a similar correlation between miRNA-122/miRNA-30a and IL-17A was observed, suggesting a general pathophysiological mechanism for COPD induction and maintenance. Together, these findings indicate that decreased miRNA-122 and miRNA-30a may represent possible markers of COPD worsening. In accordance with these results, Liu et al. established the importance of the MALAT1/miR-30a-5p/JNK signaling pathway in the pathogenesis of COPD. This study revealed that the expression of miR-30a-5p was reduced in human bronchial epithelial cells exposed to cigarette smoke extract and in COPD mouse lungs due to the sponge action of the long non-coding RNA MALAT1, which promoted airway inflammatory response via facilitating the phosphorylation of JNK [65].

We have preliminary evidence indicating that miRNA-1246 is upregulated in sputum obtained by spontaneous expectoration from exacerbated COPD patients compared to stable patients. As quantification was performed on the supernatant of centrifuged sputum, the measurements most likely reflect extracellular miRNAs, including both exosomal and cell-free fractions. miRNA-1246 is known to mediate pulmonary endothelial cell apoptosis in vitro and to promote acute lung injury in mouse models [66]. Additionally, circulating miRNA-1246 levels are increased in COPD patients with lung cancer compared to COPD patients without lung cancer [67]. Our preliminary observation suggests that the levels of miRNA-1246 are inversely correlated with the content of pathological elements of sputum. With worsening disease, elements such as proteins, biological polymers, and mucin increase in sputum, as occurs in cystic fibrosis patients [68,69]. We have developed a novel technique to evaluate the amounts of pathological substances using low-field nuclear magnetic resonance (LF-NMR) [70]. LF-NMR can measure the spin–spin relaxation time, *T*_2*m*_, of water hydrogens present in sputum. The *T*_2*m*_ value depends on the amounts of different pathological substances in sputum and is inversely correlated with their abundance. Our preliminary data indicate an inverse correlation between miRNA-1246 and *T*_2*m*_ in the sputum of COPD patients. This opens the possibility that miRNA-1246 may be a novel biomarker of COPD worsening able to reflect variations in the biophysical characteristics of sputum, thus better capturing COPD severity. Finally, we are developing an algorithm to integrate and facilitate the interpretation of miRNA levels in conjunction with the biophysical properties of sputum, as assessed by *T*_2*m*_. This approach is expected to support and streamline its implementation in routine clinical practice.

**Table 2:** Quantification of sputum miRNAs in COPD by qPCR.

miRNA	Expression/ Clinical Utility	Sample	Cohort	Sex (M/F)	Age (y)	Ref
miR-155	↑ (smokers)/ Severity	Sputum whole	COPD (n = 40) Controls (n = 20)	24/16 10/10	67.6 57.7	[41]
miR-21 miR-223 miR-155 miR-126 miR-146a	↑/Diagnosis, Severity, Exacerbation ↑/Diagnosis, Severity ↓/Diagnosis, Severity ↓/Diagnosis, Severity ↓/Diagnosis, Severity	Sputum-derived exosome	COPD (n = 20) Controls (n = 10)	19/1 9/1	70.0 65.0	[45]
miR-338 miR-145	↑/Diagnosis, Severity ↑/Diagnosis, Severity	Sputum (exosome and cell-free) serum	COPD (n = 31) Controls (n = 7)	Na/Na Na/Na	70.3 65.2	[52]
let-7c miR-125b	↓ (smokers) /Severity ↓ (smokers) /Severity	Sputum (exosome and cell-free)	COPD/ smokers (n = 12) Smokers (n = 10) Never-smokers (n = 10)	10/2 6/4 5/5	63.2 53.3 53.9	[54]
miR-181a	↑/Severity	Sputum (exact source not defined)	COPD (n = 20) Controls (n = 20)	9/11 9/11	56.5 56.4	[58]
miR-122 miR-30a	↓/Exacerbation ↓/Exacerbation	Sputum (exact source not defined) plasma	Exacerbated (n = 16) Stable COPD (n = 22)	10/6 12/10	63.8 62.4	[63]
miR-1246	↑/Exacerbation	Sputum (exososome and cell-free)	COPD Controls	Na Na	Na Na	Unpub results

Abbreviations: COPD, chronic obstructive pulmonary disease. ↑ increased amount, ↓ decreased amount, Na: non applicable.

## 3. Conclusions

### 3.1. General Consideration

The quantification of miRNAs in COPD sputum represents a relatively recent approach to disease monitoring, which likely explains the limited number of studies found in the literature. Nevertheless, this field holds significant potential for improving COPD stratification and patient monitoring.

Overall, the collected evidence highlights a complex but increasingly consistent role of miRNAs present in the sputum in COPD monitoring. Several miRNAs, including miRNA-21, miRNA-30a, miRNA-122, miRNA-126, miRNA-145, miRNA-146a, miRNA-155, miRNA-181a and miRNA-338, are differentially expressed in the sputum of COPD patients, reflecting distinct aspects of inflammation, immune dysregulation, airway remodeling, and disease exacerbation. Although some discrepancies are observed across studies, these differences can largely be attributed to methodological variability, limited cohort sizes, and population heterogeneity.

Importantly, the studies presented converge on the concept that sputum represents a highly informative and non-invasive biological matrix for assessing local airway inflammation. In contrast to circulating miRNAs, sputum-derived miRNAs may more accurately reflect lung-specific pathological processes, making them particularly suitable for disease monitoring and stratification. The combination of multiple miRNAs, rather than single markers, appears to significantly improve diagnostic accuracy and the ability to distinguish disease stages.

Emerging data, including preliminary findings on miRNA-1246 and its inverse correlation with LF-NMR-determined sputum properties, further support the potential of integrating molecular and biophysical biomarkers to better capture COPD severity and progression. This multimodal approach may ultimately enable the identification of robust biomarker combinations capable of predicting exacerbations, monitoring disease progression, and guiding therapeutic decisions, paving the way toward more personalized management of COPD.

### 3.2. Technical Considerations

Future research should move toward a more integrated approach, with particular attention to both methodological rigor and translational potential. In this regard, it will be essential to conduct large multicenter studies involving well-characterized patient cohorts, defined according to standardized criteria such as GOLD stages, smoking status, and comorbidities. Cross-study discrepancies are amplified by differences in patient cohorts and analytical platforms. Studies often involve relatively small and heterogeneous populations, with variability in disease severity, smoking status (current vs. former smokers), treatment regimens (e.g., corticosteroids), and the presence of comorbidities such as asthma or cardiovascular disease. These factors can independently influence miRNA expression profiles, potentially confounding disease-specific signals. Additionally, demographic variables, including age, sex, and environmental exposures, are not always consistently controlled. Moreover, future investigations should ideally adopt longitudinal designs, allowing researchers to monitor changes in miRNA expression over time and better understand their relationship with disease progression.

Efforts need also to be directed toward improving consistency across studies by establishing standardized protocols for sputum collection, processing, and storage. The lack of standardized protocols for sputum collection and processing remains a major source of variability across studies. Induced versus spontaneous sputum, differences in induction protocols (e.g., hypertonic saline concentration and duration), and variable handling procedures (such as the use of dithiothreitol for mucus digestion) can all significantly affect the cellular and extracellular composition of sputum samples. Moreover, inconsistencies in the selection of sputum fractions, i.e., whole sputum, supernatant, cell pellet, or extracellular vesicles, can lead to different miRNA profiles. Pre-analytical factors, including time to processing, storage temperature, and freeze–thaw cycles, further contribute to RNA degradation and variability. Without harmonized and rigorously validated protocols, these factors may introduce substantial noise that complicates cross-study comparisons and may obscure biologically meaningful signals.

Harmonization of miRNA quantification techniques and normalization strategies, together with the definition of shared reference controls, will be crucial to ensure comparability and reproducibility of results. Considerable heterogeneity exists in miRNA extraction, quantification, and normalization workflows. Different RNA isolation kits, extraction efficiencies, and input volumes can influence miRNA yield and purity, leading to discrepancies in downstream analyses. Similarly, diverse quantification platforms can influence the final output. One of the most critical unresolved issues is normalization: while some studies rely on endogenous controls, others use exogenous spike-ins or global mean normalization, each with inherent limitations. The absence of accepted reference genes for sputum miRNA analysis further complicates interpretation, as commonly used controls may themselves vary under inflammatory conditions. This methodological heterogeneity undermines reproducibility and makes it difficult to define robust and comparable expression thresholds.

## 4. Future Perspectives

Translating sputum-derived miRNA research from bench to bedside will require a coordinated effort to bridge technical innovation, clinical validation, and healthcare integration. While current findings are promising, several strategic developments are necessary to fully unlock their clinical utility.

One of the most important priorities is the development of standardized, clinically validated assays. For sputum miRNAs to become part of routine diagnostics, robust and reproducible platforms must be established with clearly defined performance metrics, including sensitivity, specificity, and inter-laboratory reproducibility. Regulatory approval will depend on well-designed validation studies demonstrating clinical utility across diverse patient populations. The establishment of reference ranges and clinically actionable thresholds will be essential to enable interpretation by clinicians and integration into existing diagnostic workflows.

The advancement of point-of-care and minimally complex testing platforms represents another critical translational step. Current miRNA analysis methods often rely on sophisticated laboratory infrastructure and specialized personnel, limiting their applicability in routine clinical settings. Emerging technologies, such as portable PCR devices and biosensor-based detection platforms, may offer the potential to simplify workflows, reduce turnaround times, and lower costs. The development of rapid, sputum-based miRNA assays could enable real-time clinical decision-making, particularly in the context of exacerbation risk assessment and treatment monitoring.

Integration with multimodal clinical data is expected to significantly enhance clinical impact. Sputum miRNA profiles may not be considered in isolation but rather combined with spirometric data, imaging findings, symptom scores, and other biomarkers (e.g., blood eosinophils, inflammatory mediators). The incorporation of such multidimensional datasets into unified predictive models, supported by machine learning and artificial intelligence, could enable more precise phenotyping and risk stratification of COPD patients.

Longitudinal and interventional studies will be important for demonstrating true clinical value. Beyond cross-sectional associations, future studies have to show that miRNA-based biomarkers can meaningfully inform clinical decisions, improve outcomes, or reduce healthcare burden. For example, studies should evaluate whether miRNA-guided interventions can predict or prevent exacerbations, guide therapy selection (e.g., corticosteroid responsiveness), or monitor treatment efficacy over time. Demonstrating such utility will be critical for adoption by clinicians and inclusion in clinical guidelines.

Personalized medicine applications represent a particularly promising avenue. Given the well-recognized heterogeneity of COPD, sputum miRNA signatures could help define biologically distinct COPD types, enabling targeted therapeutic strategies. In the future, patient-specific miRNA profiles may inform individualized treatment plans, optimizing drug selection and minimizing unnecessary interventions. This approach aligns with the broader movement toward precision medicine in respiratory diseases.

Finally, economic and implementation considerations must not be overlooked. For widespread adoption, sputum miRNA assays have to demonstrate cost-effectiveness compared to existing diagnostic and monitoring strategies. Health system integration will require streamlined workflows, clinician training, and clear clinical guidelines. Collaboration between researchers, clinicians, industries, and regulatory bodies will be essential to ensure that technological advances translate into accessible and scalable healthcare solutions.

In conclusion, the future translational landscape of sputum miRNA research in COPD is highly promising but will depend on overcoming key methodological, clinical, and logistical barriers. With continued progress in assay standardization, technological innovation, and integrative data analysis, sputum-derived miRNAs have the potential to become a cornerstone of non-invasive, precision-based COPD management.

## Figures and Tables

**Table 1:** Advantages and limitations of sputum as a biological source for miRNA detection in COPD.

Advantages	
Non-invasive sampling	Sputum collection is non-invasive and repeatable, unlike bronchoscopy or bronchoalveolar lavage, which requires more complex procedures.
Multiparametric analysis	Suitable for biochemical, microbiological, rheological, and cellular analyses.
Early disease reflection	Reflects early local inflammatory changes and signals associated with exacerbations.
Reduced systemic confounding	Less influenced by comorbidities (e.g., cardiovascular disease, diabetes), systemic medications, and systemic inflammation compared to blood-based biomarkers.
Sensitivity to local therapies	Changes in sputum miRNAs better reflect responses to inhaled treatments (e.g., corticosteroids, bronchodilators).
Limitations	
Sample quality variability	Collection of adequate sputum may be technically challenging and not feasible in all patients.
Risk of contamination	Samples may be affected by saliva contamination and variability introduced by coughing.
Lack of standardization	miRNA quantification in sputum shows lower methodological standardization compared to blood-based assays.

## Data Availability

No new data were created or analyzed in this study. Data sharing is not applicable to this article.
